# Investigation of Regeneration Mechanisms of Aged Solar Salt

**DOI:** 10.3390/ma14195664

**Published:** 2021-09-29

**Authors:** Julian Steinbrecher, Alexander Bonk, Veronika Anna Sötz, Thomas Bauer

**Affiliations:** 1 Institute of Engineering Thermodynamics, German Aerospace Center (DLR), D-70569 Stuttgart, Germany; Alexander.Bonk@dlr.de; 2 Institute of Engineering Thermodynamics, German Aerospace Center (DLR), D-51147 Cologne, Germany; veronika.soetz@th-deg.de (V.A.S.); Thomas.Bauer@dlr.de (T.B.); 3 Research Center Modern Mobility, Technology Campus Plattling, D-94447 Plattling, Germany

**Keywords:** thermal energy storage, concentrating solar power (CSP), molten nitrate salt, thermal stability, liquid-gas reactions

## Abstract

The scope of our study was to examine the potential of regeneration mechanisms of an aged molten Solar Salt (nitrite, oxide impurity) by utilization of reactive gas species (nitrous gases, oxygen). Initially, aging of Solar Salt (60 wt% NaNO_3_, 40 wt% KNO_3_) was mimicked by supplementing the decomposition products, sodium nitrite and sodium peroxide, to the nitrate salt mixture. The impact of different reactive purge gas compositions on the regeneration of Solar Salt was elaborated. Purging the molten salt with a synthetic air (p(O_2_) = 0.2 atm) gas stream containing NO (200 ppm), the oxide ion concentration was effectively reduced. Increasing the oxygen partial pressure (p(O_2_) = 0.8 atm, 200 ppm NO) resulted in even lower oxide ion equilibrium concentrations. To our knowledge, this investigation is the first to present evidence of the regeneration of an oxide rich molten Solar Salt, and reveals the huge impact of reactive gases on Solar Salt reaction chemistry.

## 1. Introduction

Thermal energy storage with molten nitrate salts at 565 °C is currently employed in several Concentrating Solar Power (CSP) plants to provide dispatchable and renewable electricity in the MW-scale. To increase the solar-to-power conversion efficiency, elevating the maximum operating temperature is one possible strategy [1,2]. For a nitrate based molten salt storage system, chemical stability is challenged at temperatures beyond 565 °C and thus effort needs to be made to maintain thermal properties of this sensible heat storage material. The decomposition and equilibrium reactions of a nitrate-based molten salt has extensively been investigated over the past decades and a variety of decomposition mechanisms are reported [3,4,5,6]. It is widely accepted that in a first step nitrate ions (NO_3_^−^) compose to form nitrite ions (NO_2_^−^) under the release of oxygen (Equation (1)).

(1) $${\mathrm{NO}}_{3}^{-}⇌{\mathrm{NO}}_{2}^{-}+1/2O_{2}.$$

In the second step, the nitrite ion further decomposes to form oxide ions. A reaction, most commonly referred to as a possible decomposition mechanism for nitrite, is the formation of metal oxide, accompanied by the evolution of NO and NO_2_ (Equation (2)).

(2) $$2{\mathrm{NO}}_{2}^{-}⇌O^{2-}+\mathrm{NO}+{\mathrm{NO}}_{2}.$$

Beside these ions, the evolution of several reactive gas species, namely oxygen, nitrogen and nitrous oxide (N_2_O) has been discovered [7]. It has to be emphasized that the exact stoichiometry of Equation (2) has not been determined experimentally and may also depend on atmospheric composition. Additionally, at different temperatures, other reaction paths may become favorable [8,9]. Nevertheless, the influence of nitrous gases on the nitrite-oxide equilibrium becomes more tangible through Equation (2). In this regard, Sötz et al. reported the stabilization of molten Solar Salt with a purge gas flow containing nitrous gases at temperatures of 600 °C and even 620 °C in terms of stable nitrate-nitrite- and low oxide ion concentrations.

In this study, the positive effect of the stabilizing gases is tested to its limits. More specifically, the reduction of high oxide ion levels (0.15 wt% or 1 mol%) in Solar Salt-based mixtures (60 wt% NaNO_3_, 40 wt% KNO_3_) by reversing Equation (2), is demonstrated. These mixtures are artificially aged by adding unusually high concentrations of nitrite- and oxide ions (10 mol% and 1 mol%, respectively) and the effect of different purge gas compositions (20 % and 80 % O_2_, with and without 200 ppm NO) on the salt chemistry are investigated. The synthetically aged Solar Salt mixtures represent extreme conditions of decomposed Solar Salt beyond the tolerable level in practical applications. Increasing the operating temperature, the threat of salt decomposition becomes more likely and, so far, active control of reaction Equation (2) has not been sufficiently addressed in the literature. In this paper the reduction of oxide ion concentration is defined as regeneration, which is the process to convert synthetically altered Solar Salt to Solar Salt with a tolerable low level of oxide and nitrite ions. In other words, this work presents a change of perception, where decomposition products are intentionally added to the salt melt, in order to enable the investigation of a recovery/regeneration mechanism, which has not been investigated experimentally yet.

## 2. Materials and Methods

**Figure 1:**
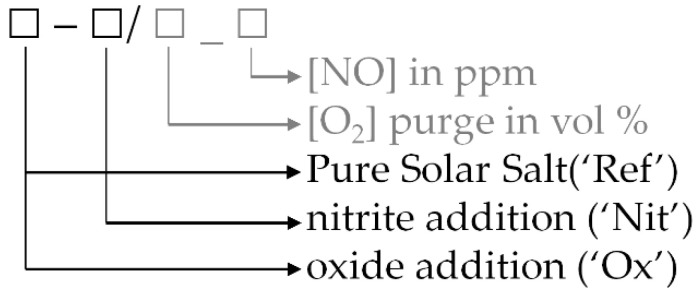
Experiment labeling according to salt composition and purge gas used.

To investigate salt regeneration, synthetically altered Solar Salt was produced by mixing NaNO_3_ (Merck, Darmstadt, Hesse, Germany, purity > 99%), KNO_3_ (Merck, purity > 99%), and Na_2_O_2_ (Merck, purity > 95%) prepared in a moisture-free atmosphere and NaNO_2_ (Merck, Darmstadt, Hesse, Germany, purity > 99%), according to the desired composition listed in Table 1. The cation content in all experiments, was fixed to the one of the Solar Salt composition (Na 65.5 mol%; K 35.5 mol%). Each of the experiments is labeled according to their salt and purge gas composition (see Figure 1).

For each experiment an autoclave test rig was loaded with 100 g of salt mix contained in an Al_2_O_3_ crucible. The autoclave test rig is temperature controlled and purged with a gas flow, and adjusted with calibrated flow meters. A detailed description of the experimental setup is given by Bonk et al. [10]. In order to remove moisture and carbon dioxide, each of the salt mixtures is heated to 120 °C for at least 24 h under a flow of synthetic air (5.0 grade, Linde Gas). Subsequently, Na_2_O_2_ is added to the salt and the desired purge gas flow (100 mL/min) is adjusted from mixing O_2_, N_2_ and a reference gas containing 1000 ppm NO in N_2_ (all 5.0 grade, Linde gas). The salt is stirred and heated (2 K/min) to the target temperature (600 °C) and the purge gas flow is adjusted according to the desired composition (see Table 1). Upon reaching 600 °C, the first salt sample is collected (t = 0 h). Over the course of the isothermal experiments (~1200 h), salt samples are extracted and analyzed to monitor salt composition (see upcoming sub-chapter for details).

To reveal the anion (and cation) content of the salt samples, ion chromatography is utilized. For ion chromatography (IC) a Metrohm model 930 Compact IC Flex is used. About 125 mg of the salt sample is dissolved in ultrapure water (500 mL, HiPerSolv, VWR, Darmstadt, Germany) and analyzed. A detailed description of the experimental procedure and calibration is given elsewhere [10]. Chromate was measured in order to monitor undesired side reactions between the steel parts and the molten salt. The limit for quantitative detection of chromate ions accounts to 0.2 mg/L or 0.05 mol% in a salt sample. Standard deviation for IC analysis was accessed via a five-fold measurement of a representative sample composition (c(NO_3_^−^) = 140.13 mg/L, c(NO_2_^−^) = 26.43 mg/L, c(CrO_4_^2−^) = 0.72 mg/L). 

To identify additional cationic species (other than sodium and potassium) arising from steel corrosion (iron, chromium, nickel), an iCE 3000 series atomic adsorption spectrometer from Thermo Fisher Scientific (Waltham, Massachusetts, USA) was utilized. In a standardized procedure, about 50 mg of the salt sample are dissolved in 50 mL of ultrapure water including 0.5 mL of 69% HNO_3_ and 0.5 mL of a 10% CsCl solution. Calibration was performed with standard solutions (Roth, certified reference material; (iron (1003.0 ± 2.2 mg/L; chromium (1000.9 ± 3.1 mg/L; nickel (1001.8±3.5 mg/L)) for iron, chromium and nickel and freshly prepared for the desired concentration range (1–5 mg/L). 

The oxide and carbonate contents are determined by inert gas acid base titration (Metrohm Titrando 800, Herisau, Switzerland). Regardless of the specific oxide ion species actually present in the melt (O_2_^−^, O_2_^2−^, O^2−^), all are summarized and expressed as O^2−^ values in this work. About 500 mg of each salt sample was dissolved in 160 mL ultrapure water and titrated with 0.01 M HCl (Titrisol standard solution). Calibration of the HCl titrant was performed by multiple titrations of a known amount of pre dried Na_2_CO_3_ (Merck, Darmstadt, Hesse, Germany, purity > 99.5%). The oxide ion concentration of the molten salt is calculated back from the measured hydroxide (2 OH^−^ converted to 1 O^2−^) and carbonate (1 CO_3_^2-^ converted to 1 O^2−^) concentrations, obtained from acid base titration [10,11,12]. The carbonates are likely to be formed after sample extraction, since the purge gases are of high purity and leak rates of the test rig are low. It is reasonable and already reported that oxide ions react with atmospheric carbon dioxide before or during titration [10,13]. In the presence of nitrite, the found carbonate ion concentration is significantly lower (10–20%) when compared to the true value. In order to take this systematic error into account, error bars of the oxide ion concentration contain the maximum error for the carbonate detection and for this reason are not symmetric. The detection limit with regard to salt composition was below 0.1 mol% O^2−^. 

For mol fractions of each ionic species all uncertainties are combined via quadratic error propagation and represented as error bars in the figures (see Tables S1–S3). Overall, the most relevant post analysis methods were IC and titration to monitor the nitrite and oxide level for the Solar Salt regeneration. Other measurements were supplementary to monitor undesired side reactions with some steel parts.

## 3. Results and Discussion

### 3.1. Behavior of an Oxide Rich Nitrate Melt under Various Conditions

In the following, changes in the anion content of the molten salt over the course of the experiment are discussed in order to reveal a potential influence of different purge gas compositions on the molten salt chemistry. In order to avoid an incorrect interpretation of the experimental results, the effect of each reaction parameter needed to be analyzed with caution. For clarity, the different experiments listed in Table 1 are categorized and discussed separately in a twofold manner. To exclude the effect of the nitrate–nitrite equilibrium, for the first set the results of molten salt purged with p(O_2_) = 0.2 atm are discussed. In the second step, experiments with a purge gas containing p(O_2_) = 0.8 atm are discussed. 

**Figure 2:**
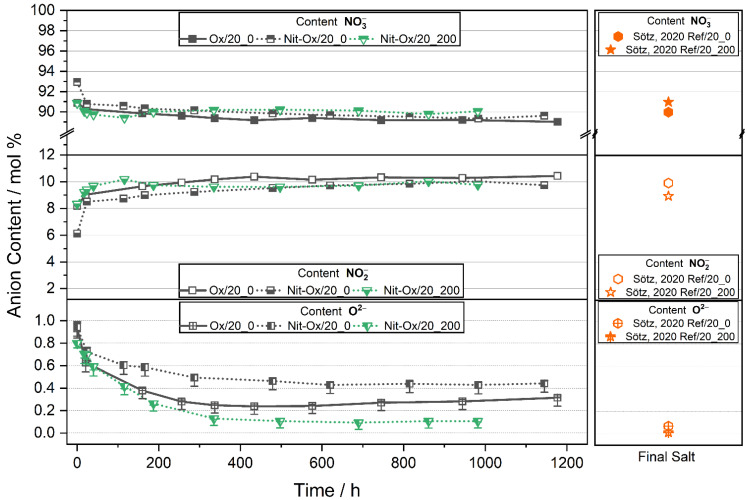
Content of nitrate, nitrite and oxide ions of investigated nitrate melts in this work with oxide addition. Literature data (orange symbols) shown (Ref/20_0, Ref/20_200) without oxide addition. All melts were stored under synthetic air with/without 200 ppm NO indicated by the label. Error bars not visible are within symbol size.

The summarized data of experiments performed under p(O_2_) = 0.2 atm expressed as anion concentrations over time, is illustrated in Figure 2. Reference experiments of pure Solar Salt already published are marked orange and named according to the applied purge gas (Ref/20_0, Ref/20_200) [7]. Within 200 h, all experiments exhibited a steady concentration of nitrates and nitrites. The molar fraction of nitrate stabilized between 89.0, 89.3, 89.6 (±0.1) mol% (Ox/20_0, Nit-Ox/20_200, Nit-Ox/20_0). Similarly, the nitrite concentration equilibrated at 10.4, 9.8, 9.7 (±0.1) mol% respectively. In general, these results agree with previously reported Solar Salt equilibrium data [7,14,15,16]. 

The oxide ion concentrations of the molten salt samples (Ox/20_0, Nit-Ox/20_200, Nit-Ox/20_0; Figure 2) decreased steadily during the first 400 h, before they stabilized at 0.3, 0.1 and 0.4 mol%, respectively. It is in contrast to our expectation that the oxide ion concentrations decrease if no NO-gas is present in the purge gas. Further salt analysis, however, indicated that the oxide ions were consumed in a concurrent reaction with the stirrer (see next chapter). At a later stage of the experiments, the oxide ion concentrations began to diverge. For molten salt without NO purge (Ox/20_0), the oxide content started to increase again after 400 h. This process was not observed for Nit-Ox/20_0, where the oxide ion content did not drop as low as in the Ox/20_0 experiment and remained constant at 0.44 (+0.01 − 0.02) mol%. Molten salt purged with 200 ppm NO (Nit-Ox/20_200) comprised about a tenth of its initial oxide concentration after 400 h, approximately 0.11 (+0.01 − 0.02) mol% after t = 1125 h.

These findings allow for some remarks on the interaction of the gas phase and the oxide reaction equilibrium. The nitrate-nitrite equilibrium (Equation (1)) is most probably not directly affected by the presence of nitrous gas, and thus equilibrium levels for nitrate and nitrite are similar to published equilibrium data without additional oxide ions (Ref/20_0). In contrast to our expectation, for experiments with the addition of nitrite (Nit-Ox/20_0, Nit-Ox/20_200) the initial nitrite ion concentration after heating to the target temperature (at t = 0 h) was below 10 mol%. Presumably this is the case due to the occurrence of catalytic reactions involving the oxide ions and the oxidation of nitrite during the heating process, as it has been reported elsewhere [17]. The results point to the probability that with the presence of oxide ions, the effect of temperature and p(O_2_) on nitrate-nitrite reactions is occurring more rapidly. As the focus of this study was on equilibrium reactions at 600 °C, we are aware that interpretations of reaction kinetics must be done with great caution and qualitatively only.

Close examination of the oxide content reveals that the molten salts in each of the three experiments follow a different reaction mechanism. For Nit-Ox/20_200 with nitrite and oxide addition and NO in the purge gas, the molten salt is first regenerated, as expressed by a significant reduction of the oxide ion concentrations. Subsequently, the oxide level is in equilibrium (unchanged), which can be attributed to the presence of NO in the atmosphere. It is reasonable to assume that the oxide ions are regenerated by the back reaction of Equation (2). This effect is visible in the nitrite content of Nit-Ox/20_200 having a maximum value after 116 h (Figure 2), which is not the case for molten salts purged with synthetic air (Ox/20_0, Nit-Ox/20_0). Compared to the literature experiment from Sötz et al. (Ref/20_200), the oxide ion equilibrium concentration at the end of the experiment of Nit-Ox-20_200 is somewhat higher (0.006 mol% vs. 0.11 mol%). In the case of the Ox/20_0 experiment with no NO in the gas stream, the nitrous gases evolving from the melt are constantly purged out of the crucible, resulting in p(NO) being effectively zero and therefore not allowing for any chemical equilibrium of Equation (2). The high but constant oxide ion content in Nit-Ox/20_0, is somewhat confounding, but could be explained as the result of a kinetic effect. Compared to Ref/20_0, the oxide content in our experiment is four times higher. Thus, with respect to Equation (2), nitrite decomposition is less favorable, slowed down, and the melt appears stabilized. It is possible that longer experimental durations would show an increasing oxide ion concentration of the Nit-Ox/20_0 experiment as a result of ongoing nitrite decomposition. 

**Figure 3:**
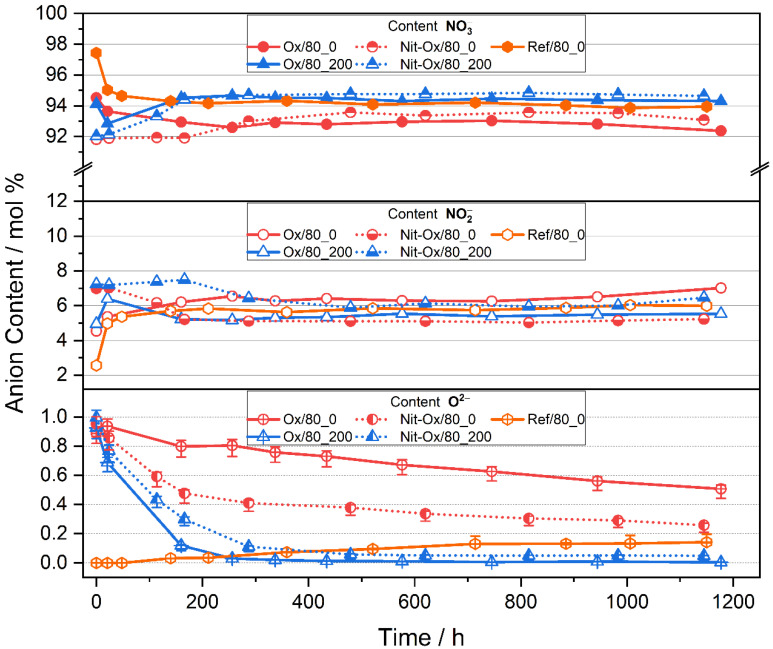
Content of nitrate, nitrite and oxide ions of investigated nitrate melts. Reference data shown (Ref/80_0) did not contain oxide dopant. Salts were stored under 0.8 atm O_2_ with/without 200 ppm NO and with/without added nitrite indicated by the label. Error bars not visible are within symbol size.

The second set of experiments was performed with oxygen rich purge gas (p(O_2_) = 0.8 atm) are reduce nitrate decomposition and consequently study its effect on the oxide equilibrium. Similar to the previously discussed set of experiments at p(O_2_) = 0.8 atm all of the experiments reach nitrate-nitrite-equilibrium with a ratio close to Ref/80_0 (Figure 3, orange). Furthermore, nitrite addition did not influence the final equilibrium values. All experiments with artificially added oxides showed a decrease of oxide concentration over time.

In the reference experiment (Ref/80_0) with pure Solar Salt (Figure 3, orange) the nitrite content increased before it reached a steady state at 6.0 (±0.1) mol%, while the nitrate content stabilized at 93.9 (±0.1) mol%. This result is consistent with thermodynamic expectations (93.3(±0.3):6.7(±0.1) mol% NO_3_^−^:NO_2_^−^ [7]) and in agreement with Equation (1), where increasing the oxygen partial pressure will push equilibrium composition to the nitrate side, thereby lowering the nitrite content. The detected oxide ion concentration increased continuously, with a final value of 0.14 (+0.01 − 0.02) mol% at 1150 h. This behavior is again explained by the fact that all gases evolving from the molten salt are purged out of the test rig, allowing no equilibration of Equation (2). 

For experiments without NO in the atmosphere and added oxides, the oxide ion content of Ox/80_0, Nit-Ox/80_0 decreased over the course of the experiments, mainly due to consumption of those ions during corrosion reactions. With the appliance of a purge gas containing NO, the oxide ion content was significantly lower compared to experiments without NO. Detected values were as low as 0.05(±0.01) mol% for Nit-Ox/80_200 and below the detection limit (<0.01 mol%) for Ox/80_200. Rapid regeneration again resulted in a temporary increase of the nitrite content during the early stages of the experiments. This can be seen for experiment Nit-Ox/80_200 and Ox/80_200 after 21 and 166 h, respectively.

It is worth mentioning that the oxide ion equilibrium level under 0.8 atm O_2_ (Figure 3, Nit-Ox/80_200 with a value of 0.05 (±0.01) mol% at the end of the experiment) is about half compared to 0.2 atm O_2_ purge (Figure 2, Nit-Ox/20_200 with a value of 0.11 (+0.01 − 0.02) mol% at the end of the experiment). This demonstrates that by reducing the equilibrium content of nitrite from about 10 mol% (p(O_2_) = 0.2 atm) to 6 mol% (p(O_2_) = 0.8 atm), the equilibrium oxide ion content is also decreased. It is concluded in this work that the oxygen partial pressure indirectly controls nitrite decomposition to oxides.

### 3.2. Indicators for Corrosivity in an Oxide Rich Nitrate Melt

**Figure 4:**
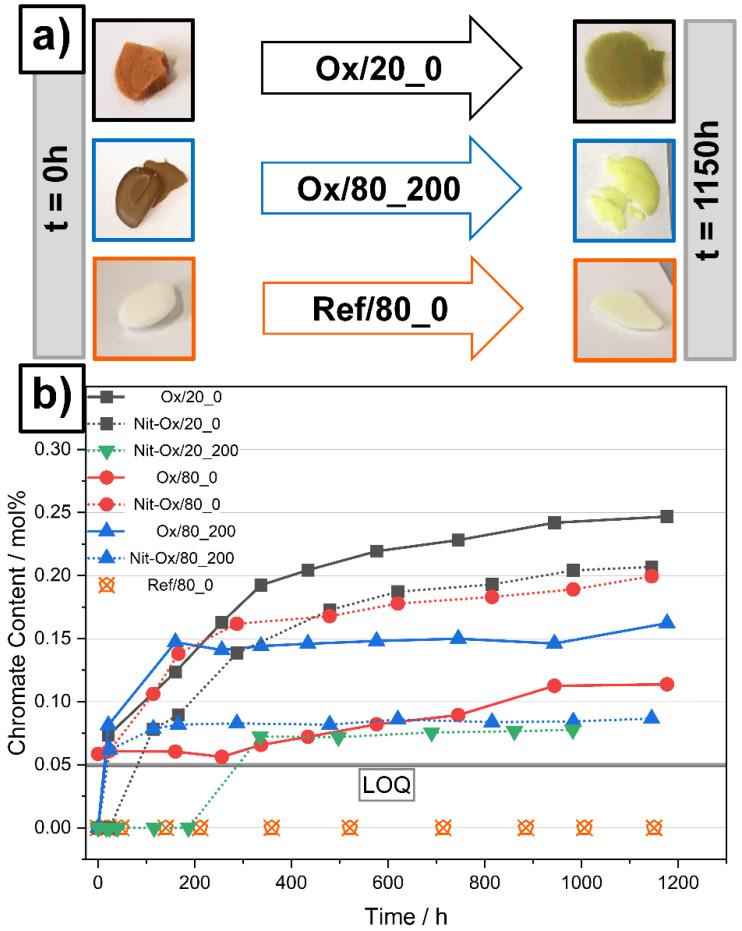
(**a**) Salt sample appearance initially (t = 0 h) and at the end of the experiments (t = 1150 h); (**b**) Chromate content over time of the molten salts. Limit of quantification (LOQ) indicated as gray bar. Reference data shown (Ref/80_0) did not contain oxide impurity. Error bars lay inside symbol.

The addition of sodium peroxide significantly altered the chemical properties of Solar Salt. In our experiments, the shaft of the stainless steel stirrer suffered from corrosion attack through the oxide ion. We utilized the detection rate of chromate as a qualitative indicator for the apparent corrosivity of our molten salts [18]. Molten salt without the addition of oxide ions (Ref/80_0) did not contain detectable chromates and the salt remained colorless over the course of the experiment. In contrast, experiments containing oxide ions showed steeply increasing chromate concentrations to approximately 0.1 mol%, within the first 200 h (Figure 4b), indicating high corrosivity compared to experiments without oxide addition. The salt samples containing even moderate concentrations of chromates exhibited a bright yellow or yellow/green color (Figure 4a).

Apart from chromium impurities, iron was also detected (up to 70 ppm) in some of the salt samples which contained oxide ions (see Table S4). By comparing the sample appearance depicted in (Figure 4a), a brown/green color (Ox/20_0, Ox/80_200) is a sign for transition metal impurities (e.g., Fe, Cr) dissolved in the salt [14,19,20]. Because corrosion reactions consume oxide ions and the investigated oxide ion regeneration process is taking place at the same time, great caution was necessary for the interpretation of oxide equilibrium data (Figure 2 and Figure 3). However, during experiments with NO purge, the chromate levels stabilized, which indicates that corrosion reactions stopped at some point (e.g., experiments Ox/80_200, Nit-Ox/80_200 and Nit-Ox/20_200) and oxide ion equilibrium data was collected. It can be concluded that the regeneration of aged molten salt suppressed the release of transition metal impurities most likely originating from corrosion reactions. An extensive corrosion study needs to be conducted in order to generate quantitative data on this effect, and that was not within the scope of this investigation.

## 4. Conclusions

In this study, we demonstrated the regeneration of a decomposed oxide ion rich molten Solar Salt towards literature known equilibrium levels with a very low concentration of oxides under synthetic air conditions and 200 ppm NO. Additionally, the regeneration under p(O_2_) = 0.8 atm lead to even lower oxide equilibrium levels and this showed the indirect influence of $p_{O2}$ on the oxide formation mechanism. Our data reveals the entire capability of the nitrous gas purge to recover an aged molten Solar Salt towards Solar Salt equilibrium composition at 600 °C in terms of anion content. On the basis of literature investigations, thermophysical properties, which are directly dependent on salt composition, were conserved [19,20]. This work gives valuable experimental data on the oxide equilibrium concentration in Solar Salt under different atmospheres. Furthermore, for storage applications at current (565 °C) and potentially higher operation temperatures (>565 °C), our study is crucial for stable operation of Solar Salt beyond the current operation temperature and innovative salt recovery strategies of decomposed Solar Salt. Additionally, we have shown that at 600 °C, molten Solar Salt with high oxide concentration significantly attacks steel components. By purging the molten salt with NO rich gas, the release of transition metal impurities potentially originating from corrosion reactions was retarded. However, given the short duration and varying steel surface quality in our experiments, caution must be applied when interpreting the data. Research into answering the question of a positive effect of NO purge and corrosion process reduction is already in progress.

## Figures and Tables

**Table 1:** List of experiments of this work (and literature). Labels are specified with regard to salt composition and purge gas composition. All salts exhibit Na/K ratios equal to that of ideal Solar Salt mixture (Na 65.5 mol %:K 35.5 mol %).

Label	Anion Content (mol %)			Purge Gas (100 mL/min)		
	Nitrate	Nitrite	Peroxide	N_2_	O_2_	NO
Ref/20_0 ^[a]^	100	0	0	80 vol%	20 vol%	0
Ref/20_200 ^[b]^	100	0	0	80 vol%	20 vol%	200 ppm
Ox/20_0	99	0	1	80 vol%	20 vol%	0
Nit-Ox/20_0	89	10	1	80 vol%	20 vol%	0
Nit-Ox/20_200	89	10	1	80 vol%	20 vol%	200 ppm
Ref/80_0	100	0	0	20 vol%	80 vol%	0
Ox/80_0	99	0	1	20 vol%	80 vol%	0
Nit-Ox/80_0	89	10	1	20 vol%	80 vol%	0
Ox/80_200	99	0	1	20 vol%	80 vol%	200 ppm
Nit-Ox/80_200	89	10	1	20 vol%	80 vol%	200 ppm

^[a,b]^ Reference experiments already published by Sötz et al. [7].

## Data Availability

The data presented in this study are available on request from the corresponding authors.

## Supplementary Materials

The following are available online at https://www.mdpi.com/article/10.3390/ma14195664/s1, Table S1: Titration error sources. Table S2: Ion chromatography error sources. Table S3: Maximum relative measuring uncertainty with respect to quadratic error propagation for molar ion content. Table S4: Iron content in nitrate salt samples after different exposure times. Images of the respective samples are presented for clarity.
