# Supplementary material for: Investigation of Regeneration Mechanisms of Aged Solar Salt
Source: Materials (Basel). 2021 Sep 29;14(19):5664. doi: 10.3390/ma14195664 (PMC8510360; doi:10.3390/ma14195664)
Supplement: Supplementary file 1 [file materials-14-05664-s001.zip › materials-1377382-supplementary.pdf]

materials

Supplementary Materials

Investigation of Regeneration Mechanisms of Aged Solar Salt

Julian Steinbrecher [1,*], Alexander Bonk [1], Veronika Anna Sötz [2,a] and Thomas Bauer [2]

[1] German Aerospace Center (DLR), Institute of Engineering Thermodynamics, D-70569 Stuttgart, Germany;
[2] German Aerospace Center (DLR), Institute of Engineering Thermodynamics, D-51147 Cologne, Germany;
[a] Present address: Research Center Modern Mobility, Technology Campus Plattling, D-94447 Plattling, Germany;
* Correspondence: julian.steinbrecher@dlr.de

Table S1. Titration error sources.

Error Titration	Error Size
Dosing (800 Dosino 20mL, Metrohm AG)	2% for 2 mL
Weighing (AG24-5, Mettler Toledo)	0.01 mg/0.1 mg
Maximum standard deviation of titer determination	0.00794 mol/L

Table S2. Ion chromatography error sources.

Error IC	Error Size
Weighing (AG24-5, Mettler Toledo)	0.01 mg/0.1 mg
Uncertainty volumetric flask (500 mL)	± 0.25 mL
Standard deviation nitrate determination	± 0.22 mg/L
Standard deviation nitrite determination	± 0.054 mg/L
Standard deviation chromate determination	± 0.001 mg/L

Table S3. Maximum relative measuring uncertainty with respect to quadratic error propagation for molar ion content.

	Nitrate	Nitrite	Chromate	Oxide
Measuring Range	4–170 mg/L	1–40 mg/L	0.2–20 mg/L	0.6–20 mg/L
Limit of Quantification	4 mg/L	1 mg/L	0.2 mg/L	0.6 mg/L
Estimation of maximum measuring uncertainty	0.3 %	0.9 %	0.5 %	+5.2 %–20 %[a]

[a] negative systematic error range is depending on [CO_3^{2-}]

Table S4. Iron content in nitrate salt samples after different exposure times. Images of the respective samples are presented for clarity.

Label	Time: 0 h	Appearance	140 h	Appearance	250 h	Appearance
**Ref/80_0**	n.d.	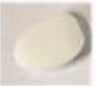	n.d.	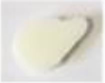	< 10 ppm (211 h)	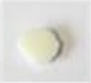
**Ox/20_0**	66 ppm	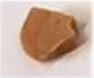	34 ppm	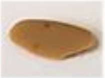	10 ppm	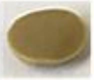
**Ox/80_0**	73 ppm	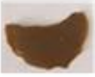	<10 ppm	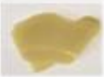	<10 ppm	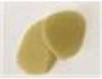
**Ox/80_200**	65 ppm	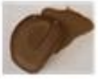	<10 ppm	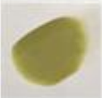	n.d.	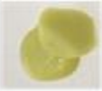
